# A Rapid Method to Immortalize Schwann Cells

**DOI:** 10.1002/pdi3.70034

**Published:** 2025-12-24

**Authors:** YanTing Zhang, Jian Zheng, Yingling Yao, Ling He, Shaoyan Liang, Guoxin Nan

**Affiliations:** ^1^ Department of Pediatric Research Institute Children's Hospital of Chongqing Medical University Chongqing China; ^2^ Stem Cell Biology and Therapy Laboratory Children's Hospital of Chongqing Medical University Chongqing China; ^3^ Chongqing Shapingba Maternity & Child Healthcare Hospital Chongqing China; ^4^ Dongguan Children's Hospital Affiliated to Guangdong Medical University Dongguan China; ^5^ Dongguan Eighth People's Hospital Dongguan China; ^6^ Dongguan Institute of Pediatrics Dongguan China

**Keywords:** immortalization, immortalized cells, improved method, SV40T antigen

## Abstract

The study of cells aids in comprehending the pathophysiology of diseases. However, obtaining a large number of primary cells in a short period is challenging, and they senesce and die after repeated passages. Therefore, establishing immortalized cell lines is necessary for conducting cellular experiments. Researchers commonly use antibiotics to screen immortalized cell models upon construction. However, due to the low transfection rate of the immortalized genes, a significant number of nonimmortalized cells are killed. The connections between the cells act as a web that floats when many cells die. As a result, successfully transfected immortalized cells are lifted and carried away, leading to only a small number of immortalized cells surviving. The surviving cells survive in the absence of other cell‐secreted factors. However, their proliferative ability is limited, which makes obtaining immortalized cell lines a time‐consuming process. This study aimed to shorten the time required to obtain immortalized cell lines by constructing immortalized Schwann cells and improving the traditional screening method. The immortalized gene transfectants were first cultured, passaged, and then screened. A comparison with the traditional screening method demonstrated the feasibility and advantages of the improved method.

## Graphical abstract

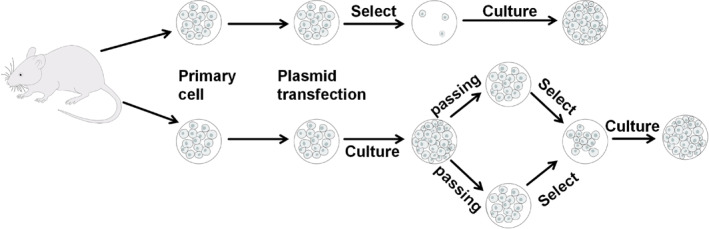

## 1 Introduction

Cells play a key role in the occurrence and development of various diseases. Various studies on cell characteristics are needed to understand disease‐related pathogenesis and pathological changes [1]. Cells can be extracted from mammalian tissues. However, the extraction of primary cells is complicated. Furthermore, the extracted cells are not pure and need to be screened with drugs or differential centrifugation [2]. Other hindrances are the difficulty in obtaining a large number of primary cells in vitro in a short period of time, the aging and death of many of the cells after multiple passages, and the heterogeneous characteristics of cells from different batches and different generations [3]. These challenges have hindered research on cells and related diseases.

When normal cells are affected by external factors during in‐vitro culture, some cells surmount the normal mechanism of death and aging. These cells have the capacity for unlimited proliferation and can be passed to many successive generations of cells. These cells are termed immortalized cells [4]. In the process of in‐vitro culture, immortalized cells gradually adapt to the growth environment. The growth rate tends to be stable, cell passage is convenient, and the phenotype does not change [5]. The unlimited proliferation capacity, homogeneity, and ability to clone permits the use of these cells for the stable expression of foreign genes. Thus, the establishment of immortalized cell lines is valuable for studying the molecular mechanisms of related diseases.

In addition to spontaneous immortalization, cell immortalization methods include virus transfection, telomerase activation, and oncogene transfection. The most common method of cell immortalization is overexpression of the monkey kidney virus SV40T antigen or the human telomerase reverse transcriptase (*hTERT*) gene in cells infected with recombinant lentiviruses [6]. The transfection of primary cells with the SV40T antigen carried by a plasmid and selection via antibiotics are common, but this method has several shortcomings. The transfection rate of the SV40T antigen is low. Furthermore, when transfection is followed by antibiotic selection, many of the nonimmortalized cells that have not been successfully transfected die because they lack a resistance gene to the antibiotic. These dead cells can be shed and float on the culture medium. Since extensive cell‐to‐cell junctions are formed, with cells woven into a web [7], the loss of these cells can also include successfully transfected and viable immortalized cells. This can drastically reduce the recovery of viable immortalized cells. Furthermore, the mutual trophic effect between surviving cells can limit their proliferative capacity due to the lack of effect of other cell‐secreted factors [8]. Consequently, obtaining stable immortalized cell lines can take longer.

Given that immortalized cell lines are capable of continuous serial passage whereas non‐immortalized primary cells tend to undergo progressive senescence and eventual elimination after a limited number of passages, the proportion of immortalized cells in the culture will gradually increase, while that of non‐immortalized cells will correspondingly decrease as the passage number increases. The immortalized cells constitute the majority of the population obtained by antibiotic selection. These surviving immortalized cells will markedly increase in size and will not be eliminated as nonimmortalized cells are exfoliated.

Therefore, in this study, we modified the traditional methods. For this study, we chose a peripheral nerve cell, Schwann cell, to test the feasibility of our modified approach to the selection process during the production of immortalized cells. Briefly, after the immortalized cell model is constructed, the cells are first cultured and passaged, and after a greater number of immortalized cells are observed by fluorescence, antibiotic selection is then performed, enabling rapid access to immortalized Schwann cell lines. Moreover, these cell lines retained most of the characteristics of primary cells.

## 2 Materials and Methods

### 2.1 Animals and Materials

Four female and two male Sprague‒Dawley rats with body masses of 220–250 g were purchased from Beijing Huafukang Biotechnology Co. Ltd. (Beijing, China) (animal certificate number: SCXK [JING] 2019‐0008). One male and two females were housed together in a cage for the purpose of feeding and mating. The rats had free access to food and water. Newborn rats were collected at 1 day of age. A total of 63 neonatal rats were utilized to complete all the experiments. All animal experiments were performed in accordance with the “Measures for the Management of Laboratory Animals in China” and the “Guiding Opinions on the Kind Treatment of Laboratory Animals” and were approved by the Ethical Review Committee for Laboratory Animal Welfare of the Children's Hospital of Chongqing Medical University (IACUC Issue No: CHCMU‐IACUC20230117002).

The complete medium for Schwann cell culture was Dulbecco's modified Eagle's medium (DMEM; Gibco, Grand Island, NY, USA) supplemented with 10% fetal bovine serum (FBS; Gibco, Grand Island, NY, USA), hereafter designated 10% FBS‐DMEM. Pancreatin (0.25%; Bioagrio, Co. Ltd., Shanghai, China) was used to digest the animal tissues.

### 2.2 Isolation and Culture of Primary Schwann Cells

Newborn Sprague‒Dawley rats were killed by cervical dislocation. The rats were sterilized by soaking in alcohol. The bilateral sciatic nerves were extracted under sterile conditions with micro tweezers and placed in DMEM. The nerve adventitia on the surface of the sciatic nerve was removed, cut with ophthalmic scissors, transferred to 3 mL of 0.25% pancreatin, and digested at 37°C for 6 h. An equal volume of 10% FBS‐DMEM was added. The filtrate was collected and centrifuged for 5 min at 1500 rpm. The supernatant was discarded, and 10% FBS‐DMEM was added. A predetermined volume of the cell suspension was inoculated in a 10 cm culture dish, which was incubated at 37°C in a 5% CO_2_ incubator. After 24 h, the medium was replaced with 10% FBS‐DMEM containing 5 μg/mL cytarabine (MedChemExpress, Monmouth, NJ, USA) to eliminate fibroblasts. After 24 h, the medium was replaced with 10% FBS‐DMEM for the culture of relatively pure primary Schwann cells at 37°C in a 5% CO_2_ incubator. The medium was replaced every 3 days.

### 2.3 Establishment of Immortalized Schwann Cells

Second‐generation Schwann cells were selected and inoculated in a 6 cm‐diameter petri dish. The cells were randomly divided into two groups (A and B). Retroviruses packaged with HEK293 immortalized human embryonic kidney cells were used to infect Schwann cells. The preparation contained the immortal gene *SV40Tag*, the hygromycin resistance gene, and the green fluorescent protein (*GFP*) gene. Twenty‐four hours after transfection, the medium was replaced with plasmid‐free 10% FBS‐DMEM, and the culture was continued, with half of the volume replaced every 3 days.

In group A, the culture medium was changed 24 h after infection. Approximately 2–3 days after seeding, the cells can proliferate to 90% confluence and are passaged at a ratio of 1:2, with medium changes performed every 2 days. The culture medium was passed continuously for six to seven generations. After 7 days of screening, the culture medium containing 0.1 g/L hygromycin was replaced with culture medium without hygromycin. At days 1, 2, 4, 7, 10, 12, 14, 16, and 18 after transfection, the number of green fluorescent cells was determined by fluorescence microscopy and fluorescence quantitative analysis was performed by Image J software (NIH, Bethesda, MD, USA).

Group B was directly screened with culture medium containing 0.1 g/L hygromycin 24 h after transfection, and the medium was replaced with culture medium without hygromycin for 7 days. After hygromycin screening, the cell morphology was observed via light microscopy.

### 2.4 Real‐Time Quantitative Polymerase Chain Reaction (RT‐qPCR)

The tenth generation of group A immortalized Schwann cells and group B primary Schwann cells were collected. RNA was extracted via an RNA extraction kit (Accurate Biology, Changsha, Hunan, China). cDNA was obtained via reverse transcription of 2 g of RNA. The primer sequences are shown in Table 1. Glyceraldehyde 3‐phosphate dehydrogenase (*GAPDH*) was used as an internal reference and standardized template to measure the level of gene expression. The reaction system was composed of 0.8 μL of cDNA, 5 μL of SYBR, 3.4 μL of double‐distilled water, and 0.8 μL of primer (the upper and downstream primers were mixed with 5 and 90 μL of double‐distilled water, respectively). The reaction conditions were 41 cycles of 95°C for 3 min, 95°C for 5 s, 60°C for 30 s, and 72°C for 30 s and a melting curve of 65°C–95°C with an increase of 0.5°C every 5 s. The experiment was repeated three times.

**TABLE 1:** Primer sequences for RT‒qPCR.

Genes	Sequences of primers (5′ → 3′)		
	Forward	Reverse	Accession number
*NGF*	GAGCGCATCGCTCTCCT	TGTACGCCGATCAAAAACGC	M36589
*BDNF*	ATAATGTCTGACCCCAGTGCC	AACCCGGTCTCATCAAAGCC	D10938
*GAPDH*	TGGAGTCTACTGGCGTCTT	GCTGACAATCTTGAGGGAG	AB017801

Abbreviations: *BDNF*, brain‐derived neurotrophic factor; *GAPDH*, glyceraldehyde 3‐phosphate dehydrogenase; and *NGF*, nerve growth factor; RT‐qPCR, real‐time quantitative polymerase chain reaction.

### 2.5 Reverse Transcriptase‒Polymerase Chain Reaction (RT‒PCR)

The obtained cDNA was amplified via RT‒PCR. The primer sequences are presented in Table 2. The reaction conditions were 35 cycles of 98°C for 5 min, 95°C for 30 s, 55°C for 30 s, and 72°C for 2 min, followed by 7 min at 72°C and finally 4°C. The PCR products were analyzed via agarose gel electrophoresis and ethidium bromide staining.

**TABLE 2:** Primer sequences for RT‒PCR.

Genes	Sequences of primers (5′ → 3′)			
	Forward	Reverse	Tm	Accession number
*SV40Tag*	AAAAAAGAATTCATGGATAAAGTTTTAAACAGAGAGG	AAAAAAAAGCGGCCGCTTATGTTTCAGGTTCAGGGG	55	NC_001669

Abbreviation: RT‐PCR, reverse transcriptase‐polymerase chain reaction.

### 2.6 Western Blot Analysis

RIPA lysis buffer (Bioagrio) containing phenylmethylsulfonyl fluoride as a protease inhibitor (Beyotime Biotechnology, Shanghai, China) was used to extract proteins from the 10th generation of group A and B immortalized cells as well as primary cells. The proteins were separated via 10% sodium dodecyl sulfate‒polyacrylamide gel electrophoresis (SDS‒PAGE) and electrotransferred to nitrocellulose membranes. Each membrane was incubated with a solution of Tris‐buffered saline‐Tween (TBS‐T) containing 5% skim milk powder (50 mM Tris‐HCl, pH 8.0, and 0.05% Tween 20) at room temperature for 1.5 h. The membranes were then incubated overnight at 4°C with one of the following primary antibodies: anti‐SV40T‐Ag (1:1000, Santa Cruz Biotechnology, Dallas, TX, USA), anti‐*NGF* (1:1000; Zen‐Bio, Chengdu, Sichuan, China), anti‐*BDNF* (1:1000; Zen‐Bio, Chengdu, Sichuan, China), anti‐P75NTR (1:1000, Proteintech, Rosemont, IL, USA), anti‐SOX10 (1:1000, Proteintech, Rosemont, IL, USA), or anti‐β‐Tublin (1:10000; Zen‐Bio, Chengdu, Sichuan, China), or anti‐*GAPDH* (1:10000, Proteintech, Rosemont, IL, USA). Each membrane was washed with TBS‐T and incubated with goat anti‐rabbit IgG (1:10000; Zen‐Bio, Chengdu, Sichuan, China) at room temperature for 1.5 h. The surface of the nitrocellulose membrane was exposed to diaminobenzidine chromogenic solution and photographed.

### 2.7 Immunofluorescence

Second‐generation Schwann cells were inoculated in confocal culture dishes at a density of 4 × 103 cells/well. When the cells reached 60% confluence, the medium was removed, and the cells were fixed by the addition of 4% paraformaldehyde for 15 min. The cells were permeabilized by the addition of 0.3% Triton‐X‐100 for 15 min, followed by the addition of 5% FBS (Gibco, Grand Island, NY, USA) for 30 min. Anti‐S100 antibody (Zen‐Bio, Chengdu, Sichuan, China) diluted 1:100 was added, and the mixture was incubated overnight in a wet box at 4°C. The secondary antibody (goat anti‐rabbit IgG; 1:1000, Zen‐Bio, Chengdu, Sichuan, China) was added, followed by incubation at room temperature in the dark for 1.5 h. The nuclei were stained at room temperature for 15 min with 5% 4′,6‐diamidino‐2‐phenylindole (DAPI; Beyotime Biotechnology, Shanghai, China). Each step was preceded by washing with phosphate‐buffered saline (PBS) three times for 5 min each. Then, an appropriate amount of antifluorescence quencher was added to the center of the confocal petri dish and incubated in the dark prior to confocal fluorescence microscopy. The expression of P75 neutrophin (*P75NTR*) and *SOX10* in group A, group B, and Schwann cells was detected via the same method.

### 2.8 Crystal Violet Staining

Primary cells and immortalized cells in groups A and B were inoculated in 24‐well plates, with three replicate wells in each group. After 48 h of cell adhesion, the medium was removed, the adherent cells were washed three times with PBS and fixed with 4% paraformaldehyde for 20 min, and 500 μL of crystal violet staining solution was added to each well. The wells were stained for 25 min, washed three times with PBS, and observed via optical microscopy.

### 2.9 Cell Proliferation

Fifteen generations of immortalized Schwann cells and Schwann cells in groups A and B were inoculated (5000 cells/well) in 96‐well plates, with three replicates for each group and five control wells without cells. Cell Count Kit‐8 (CCK‐8) reagent was added 24, 48, and 72 h after the cells were fully attached to the wells and incubated at 37°C for 2 h. The absorbance value was measured at a wavelength of 450 mm via an enzyme marker. The data were analyzed by GraphPad software (GraphPad, San Diego, CA, USA).

### 2.10 Statistical Analysis

Statistics were analyzed via GraphPad Prism 9.3.1 (GraphPad Software, San Diego, CA). Two‐way ANOVA was used to compare differences among groups. The level of significance was set at *p* < 0.05.

## 3 Results

### 3.1 Primary Cell Culture

**FIGURE 1:**
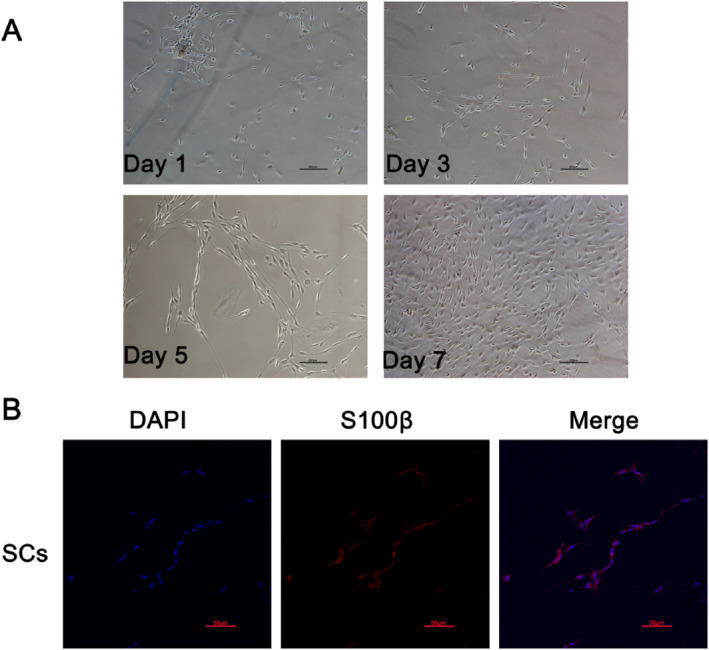
Primary Schwann cell culture and identification. (A) Cell morphology at days 1, 3, 5, and 7. The magnification is 100×, and each bar denotes 200 μm. (B) Immunofluorescence staining of primary Schwann cells (Scs) with an anti‐S100 antibody. The magnification is 200×, and each bar denotes 50 μm.

Primary Schwann cells grew adherent to the cell walls. After 7 days of culture, the cells were more elongated and formed a network or whirlpool arrangement. Immunofluorescence confocal microscopy revealed many small, fusiform, bipolar, and characteristic cell bundles. The cytoplasm of the Schwann cells was green and the nuclei were blue, indicating that the cells were positive for the *S‐100* antigen (Figure 1).

### 3.2 Immortalization of Schwann Cells

**FIGURE 2:**
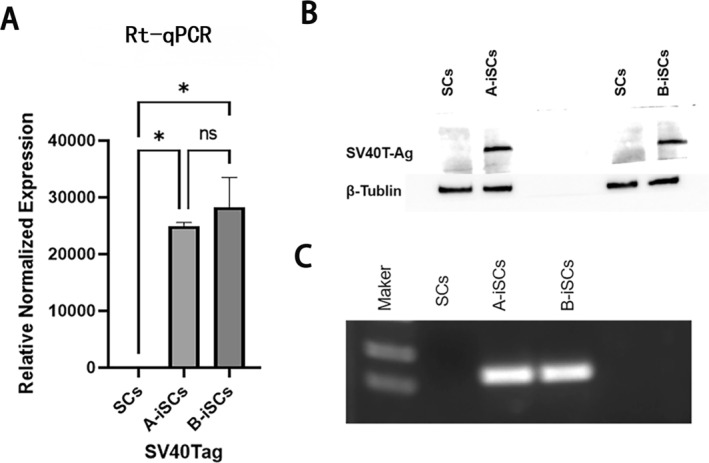
Detection of SV40T‐Ag in primary cells and ihEDMC4 cells. (A) Expression of the SV40T‐Ag gene in primary cells and immortalized Schwann cells detected by RT‐qPCR (*p* < 0.001). (B) Western blotting confirmed the expression of SV40T‐Ag in groups A and B. (C) Expression of SV40T‐Ags in Schwann cells (SCs) and immortalized Schwann cells (iSCs) analyzed by reverse transcription‐polymerase chain reaction (RT‒PCR). *means *p* 〈 0.05.

Second‐generation Schwann cells were transfected with SV40T‐Ag and divided into groups A and B, which were selected with hygromycin by different methods. Comparison of the time required for 10 consecutive passages from transfection to hygromycin screening after 7 days revealed values of 27 days for group A and 41 days for group B. The RNA and protein contents of the tenth‐generation cells and the primary cells in groups A and B were detected; SV40T‐Ag was still stably expressed in the tenth‐generation cells but not in the primary cells (Figure 2).

### 3.3 Comparison of Selection Methods

**FIGURE 3:**
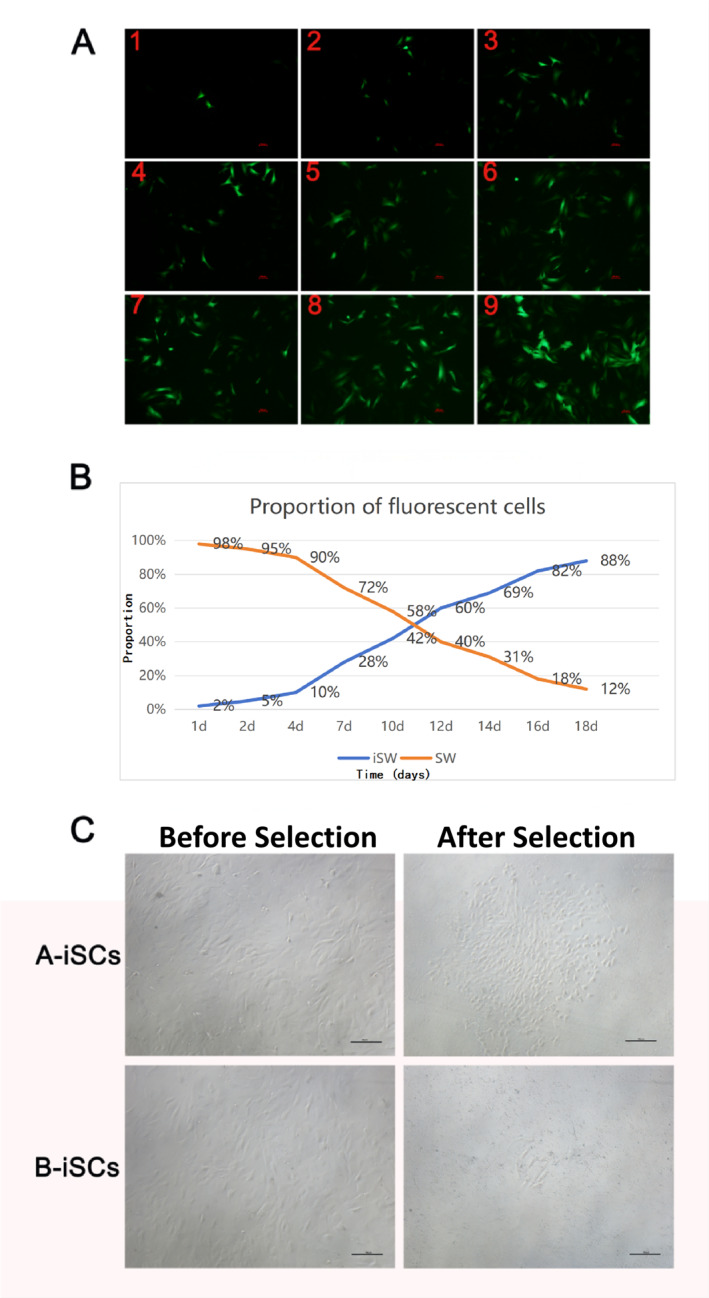
Two different selection methods. (A) Proportion of fluorescent cells in group A in different generations (please see Supporting Information S1 for details). Figures A1–A9 present the number of fluorescent cells observed via fluorescence microscopy on days 1, 2, 4, 7, 10, 12, 14, 16, and 18 after infection, respectively. (B) The proportion of immortalized cells in group A gradually increased. (C) Groups A and B were screened with hygromycin for immortalized cells. iSCs, immortalized Schwann cells; Scs, Schwann cells.

Group A was observed via fluorescence microscopy on days 1, 2, 4, 7, 10, 12, 14, 16, and 18 after infection. Passaging was carried out on days 4, 7, 10, 12, 14, and 16. With increased culture and passage times, the proportion of immortal cells increased (Figure 3A,B). Group A was selected for hygromycin resistance.

Groups A and B were screened with 0.1 g/L hygromycin for 3 days and observed via microscopy. As shown in Figure 3C, many cells remained in group A; 60%–70% of the cells survived after selection. In group B, many cells died after selection, and only a few cells survived. We compared the time taken to establish immortalized Schwann cells between groups A and B; group B took 38 days, whereas group A took 26 days, indicating a significant reduction in duration.

### 3.4 Comparison of Primary and Immortalized Cells

**FIGURE 4:**
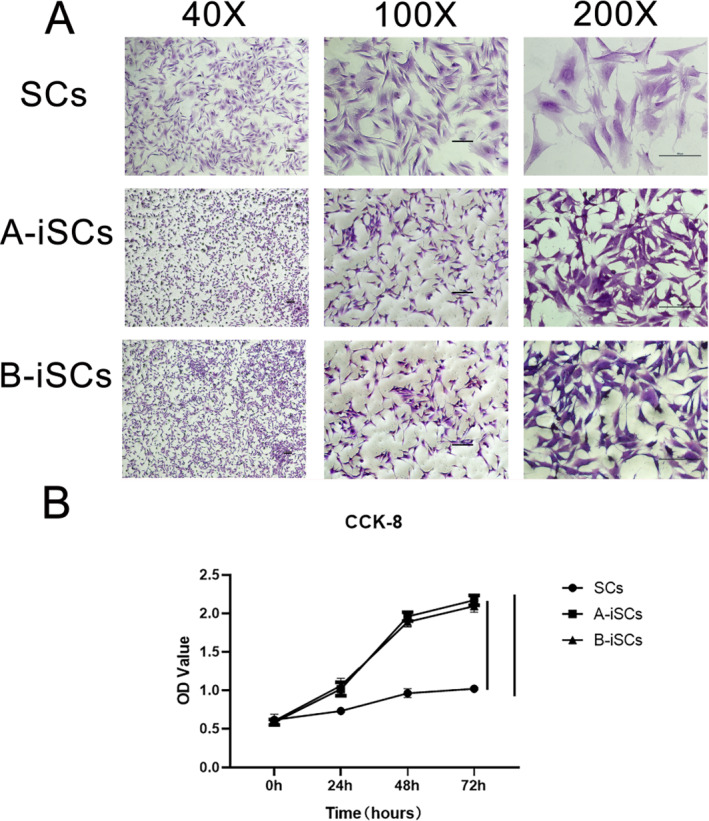
Morphology and proliferation of primary and immortalized Schwann cells. (A) CCK‐8 cell proliferation experiment results, showing the proliferation curves of immortalized cells and primary cells in groups A and B; *p* < 0.001. (B) The morphology of cells stained with crystal violet was observed via microscopy at different magnifications. iSCs, immortalized Schwann cells; OD, optical density; Scs, Schwann cells.

To compare the morphological characteristics of primary cells and immortalized cells, crystal violet was used to stain the cells. The cells were observed via microscopy. The immortalized Schwann cells had the same spindle shape as the primary cells, with elongated protrusions, mainly bipolar cells, and a few multipolar cells. However, the immortalized cells were smaller than the primary cells, and the shapes of the primary cells were more elongated (Figure 4A). Determination of the proliferation rates of primary and immortalized cells via the CCK‐8 assay revealed no difference in the proliferation rate between group A and group B (Figure 4B). However, the proliferation rate of both groups was significantly greater than that of primary cells (*p* < 0.01).

**FIGURE 5:**
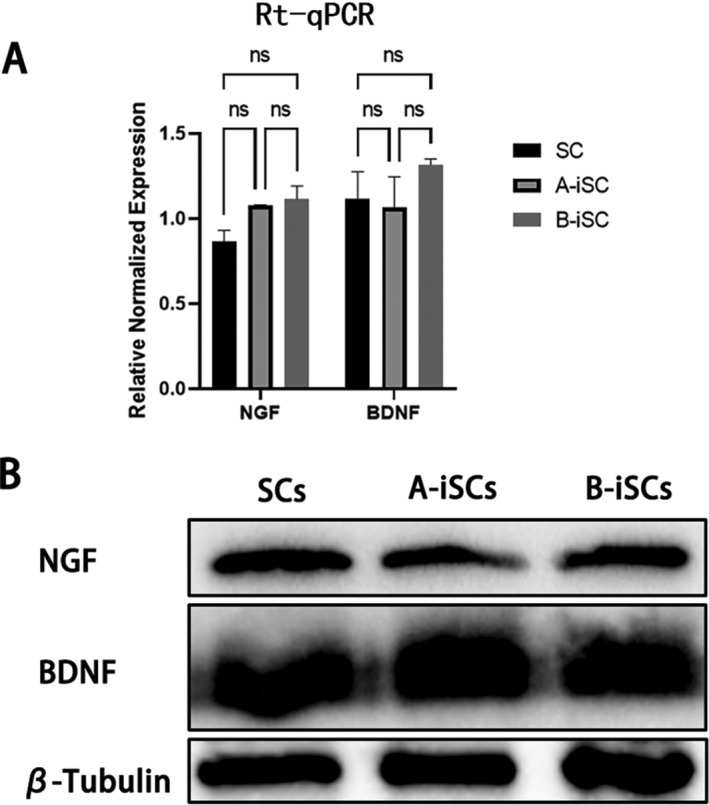
Gene expression in primary and immortalized cells. (A) Expression of nerve growth factor (*NGF*) and brain‐derived neurotrophic (*BDNF*) genes in cells analyzed by reverse transcription‐quantitative polymerase chain reaction (RT‐qPCR). (B) Western blot analysis of the protein expression of the *NGF* (32 kDa) and *BDNF* (14 kDa) genes in cells. iSCs, immortalized Schwann cells; Scs, Schwann cells.

RT‒qPCR was used to detect the expression of the *BDNF* and *NGF* genes in primary and immortalized cells. No difference in the expression of these three genes in the two cell groups was evident (Figure 5A). Western blot examination of the protein expression of these genes also revealed no difference between primary and immortalized cells (Figure 5B).

**FIGURE 6:**
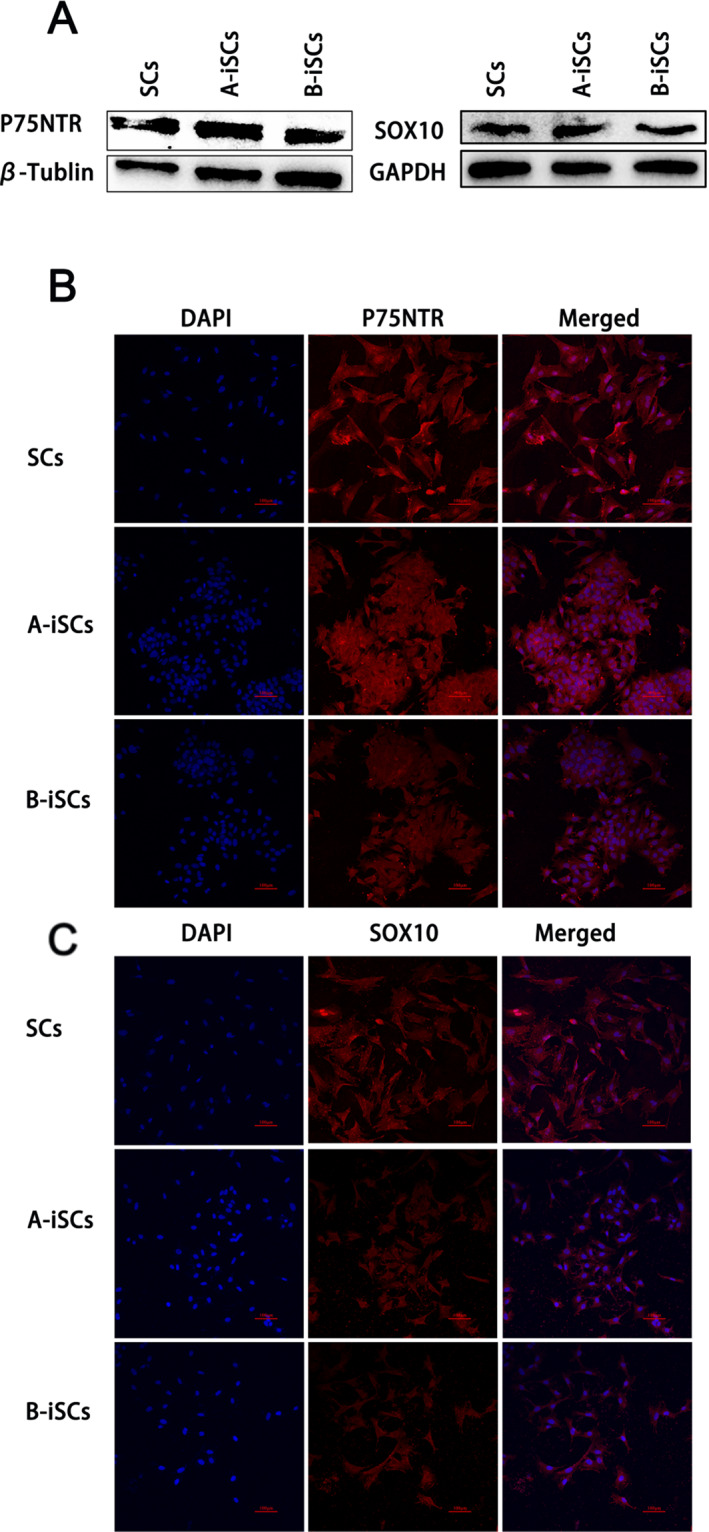
Detection of specific markers. (A) Western blot analysis of SOX10 (49 kDa) and P75NTR (44 kDa) expression in primary and immortalized cells. (B, C) Immunofluorescence staining of primary and immortalized cells. The blue marker is DAPI. The red markers in panel (B) represent P75NTR, and the red markers in panel (C) represent SOX10.

Next, to detect whether the immortalized cells expressed related specific markers of primary cells, western blotting was used to detect the expression of various markers, such as *SOX10* and *P75NTR*, which are expressed in all Schwann cells. Similarly in primary cells, the three proteins were detected in immortalized cell fluid, with no significant differences in protein expression between groups A and B (Figure 6A). The protein expression of these genes was further confirmed by immunofluorescence (Figure 6B,C), indicating that the genotypes of the primary cells and the immortalized cells were similar.

## 4 Discussion

Schwann cells are the primary glial cells of the peripheral nervous system, offering support and wrapping around nerve fibers, creating myelin sheaths, and increasing the rate of nerve signaling. This process is crucial for repairing and regenerating damaged nerves [9]. Schwann cells remove myelin debris and drive the directed growth of regenerated axons through dedifferentiation, proliferation, and migration [10]. In addition, Schwann cells produce neurotrophic factors, such as *BDNF* and *NGF*, which are necessary for neuron growth and survival [11, 12]. However, obtaining Schwann cells is difficult. The only option is to sacrifice normal nerves. Moreover, the culture of primary cells is difficult and cannot be repeated many times.

Immortalized cells continue to grow and proliferate, and this capacity can be passed on for many generations. By infecting normal cells with viruses harboring the genes of interest, genes can be specifically changed so that the cells can successfully survive the two lethal phases and develop unlimited proliferation capacity, which is the hallmark of cell immortalization [13]. There are many methods for cell immortalization. For example, human dental mesenchymal cells have been successfully immortalized via the SV40T antigen gene [14]. Another group successfully obtained immortalized human dental pulp cells by transfecting primary human dental pulp cells with retroviruses containing *hTERT* genes [15]. Many other researchers have successfully constructed immortalized cell lines via the SV40T antigen and *hTERT* genes. Collective studies have highlighted the disadvantages of the traditional methods of screening and culture. Among these disadvantages, the biggest problem is the longer cycle of immortalized cell construction. During the selection process for drug‐resistant cells, many primary cells undergo apoptosis, resulting in their suspension in the culture medium. This phenomenon may be attributed to various factors, including changes in cellular metabolism, environmental stress, and suboptimal culture conditions. The application of drugs, such as hygromycin, exerts selective pressure, which typically leads to the death of primary cells that have not been successfully transformed. Particularly in situations where there is extensive cell‒cell connectivity, multiple immortalized cells may simultaneously enter a suspended state. This phenomenon is especially pronounced in high‐density cultures, where a dense cell population can reduce interactions between cells, adversely affecting cell survival and proliferation. When cells are in suspension, they often lack the essential mutual nutritional support provided by growth factors and signaling molecules transmitted through cell‒cell contact, which may restrict their growth and proliferation. Furthermore, attached immortalized cells may experience issues, such as insufficient nutrient supply and the accumulation of metabolic waste, during the selection process, further contributing to a decreased proliferation rate [1, 16, 17].

Immortalized cells proliferate indefinitely, whereas nonimmortalized cells gradually age and are eliminated with repeated passages. In the experiment in which the *SV40Tag* gene was used to construct immortalized Schwann cells, six passages were performed after infection. It is convenient to observe changes in the number of immortalized cells by fluorescence after each cell passes. The proportions of immortalized Schwann cells at passages 1, 2, 3, 4, 5, and 6 reached 10%, 28%, 42%, 60%, 69%, and 82%, respectively. In cell culture, nonimmortalized cells are eliminated through hygromycin selection, resulting in a significant reduction in the number of dead cells within the experimental system. This selective application effectively eradicates untransformed primary cells and enhances the purity of the culture system. Moreover, the death of residual nonimmortalized cells during the culture process affects the surrounding environment, altering the distribution of nutrients and growth factors, thereby making the composition of the culture medium more favorable for the growth of surviving immortalized cells. Simultaneously, the immortalized cells situated on the walls of the culture dish established tight connections with each other, thus ceasing to be in suspension. This cell‒cell connectivity is crucial for cell growth and proliferation as it facilitates the transfer of signals and nutrients through direct contact. The relevant literature indicates that cells acquire nutrients and growth factors through contact‐dependent mechanisms, which can, to some extent, increase their proliferation rate. Therefore, retaining a greater number of immortalized Schwann cells in the culture dish not only helps increase the cell density but also leverages the synergistic interactions between these cells, significantly accelerating their growth rate. Maintaining an appropriate cell density and optimizing culture conditions are essential for the proliferation and viability of a cell line. More importantly, the immortalized Schwann cells generated via this method retained most of the characteristics of the original cells and were not significantly different from the immortalized Schwann cells constructed via traditional methods. In addition, we compared the time required to construct immortalized Schwann cells between the two methods; the original method took 38 days, and the modified method took 26 days, which was a significant reduction in time.

Western blot and immunofluorescence staining analyses revealed that markers, including *S0X10*, *S100*, and *P75NTR*, were expressed in both primary cells and the two groups of immortalized cells. These results confirmed that the generation of immortalized Schwann cells was successful. In addition, *NGF* and *BDNF* are two critical neurotrophic factors secreted by Schwann cells [18]. *NGF* and *BDNF* are important in the development and functional maintenance of peripheral nerves. Western blot and RT‒qPCR analyses revealed similar expression levels of these two key factors in primary and immortalized cells, suggesting that immortalized Schwann cells may be used in peripheral nerve research just like primary Schwann cells. During the CCK‐8 experiments, the 15th generation of iSCs was utilized and it was observed that the iSCs proliferated more rapidly than the primary Schwann cells, thereby demonstrating that the constructed iSCs could be propagated for a minimum of 14 generations. However, the morphology of immortalized Schwann cells differs from that of primary Schwann cells. Whether there are genetic changes that are unknown remains to be determined.

Overall, this study increased the number of immortalized cells by changing the method of screening the cells through multiple passages. Similarly, by increasing the number of passages, the number of primary cells is reduced, at which point many immortalized cells can be obtained by rescreening with antibiotics. By changing the screening method, we have not changed the method of constructing immortalized cells, so we believe that our improved method can be applied to other cells. This finding will have to be verified by further experiments.

## 5 Conclusion

By modifying the selection method for the preparation of immortalized cells, the immortalized Schwann cell lines obtained in the process of verifying the method can be obtained in a shorter time. These immortalized cell lines retain most of the same characteristics as the original cells do. In addition, the incorporation of the *GFP* gene into the plasmid introduced into the cells can more directly reflect the transfection efficiency and proliferation rate of immortalized cells by fluorescence microscopy. Compared with the western blot analysis or other methods, the presently derived method should prove to be more convenient for the construction of immortalized cells.

## Author Contributions

All authors contributed to the study conception and design. YanTing Zhang carried out the all experiments and drafted the manuscript. Jian Zheng, Yingling Yao and Ling He participated in the design of the study and performed the data analysis. Shaoyan Liang performed the data analysis. Guoxin Nan provides the research direction and ideas, and revised the manuscript. All authors read and approved the final manuscript.

## Funding

The authors have nothing to report.

## Ethics Statement

All animal husbandry and experimental procedures were in accordance with the “Measures for the Management of Laboratory Animals in China” and the “Guiding Opinions on the Kind Treatment of Laboratory Animals” and were approved by the Ethical Review Committee for Laboratory Animal Welfare of the Children's Hospital of Chongqing Medical University (IACUC Issue No: CHCMU‐IACUC20230117002).

## Conflicts of Interest

The authors declare no conflicts of interest.

## Significance Statement

When creating immortalized cells, researchers typically eliminate cells that were not successfully transfected with drugs. However, owing to the low transfection rate, only a small number of cells survive, which hinders the proliferation of successfully transfected cells and leads to slow cell growth. In this study, successfully transfected cells were cocultured with unsuccessfully transfected cells and passaged. After multiple passages, many immortalized cells were obtained and screened by antibiotics, resulting in an extremely large number of surviving immortalized cells. This significantly reduces the time required for cell growth.

## Supporting information


Supporting Information S1

## Data Availability

The data that support the findings of this study are available from the corresponding author upon reasonable request.
