# Supplementary material for: A Rapid Method to Immortalize Schwann Cells
Source: Pediatr Discov. 2025 Dec 24;3(4):e70034. doi: 10.1002/pdi3.70034 (PMC12753021; doi:10.1002/pdi3.70034)
Supplement: Supplementary file 1 — Supporting Information S1 [file PDI3-3-e70034-s001.pdf]

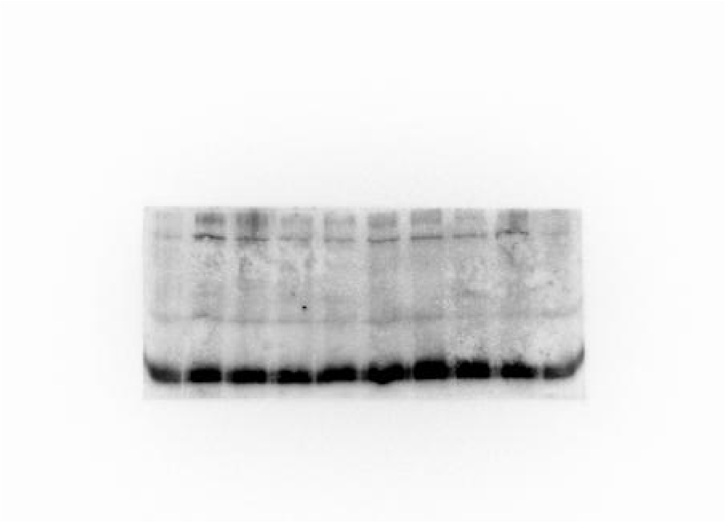
BDNF

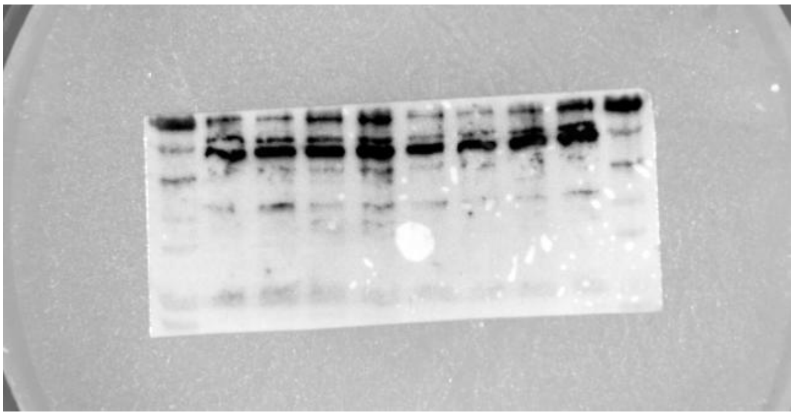
β -Tubulin

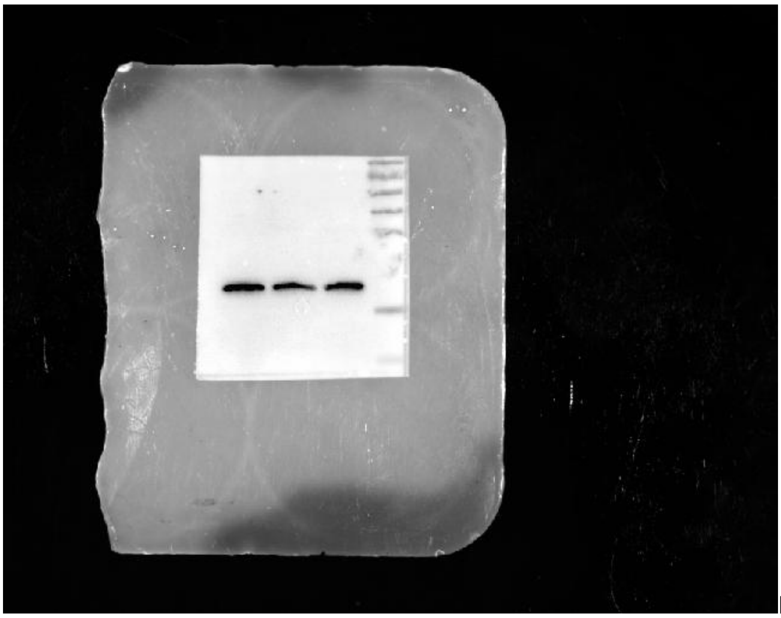
NGF

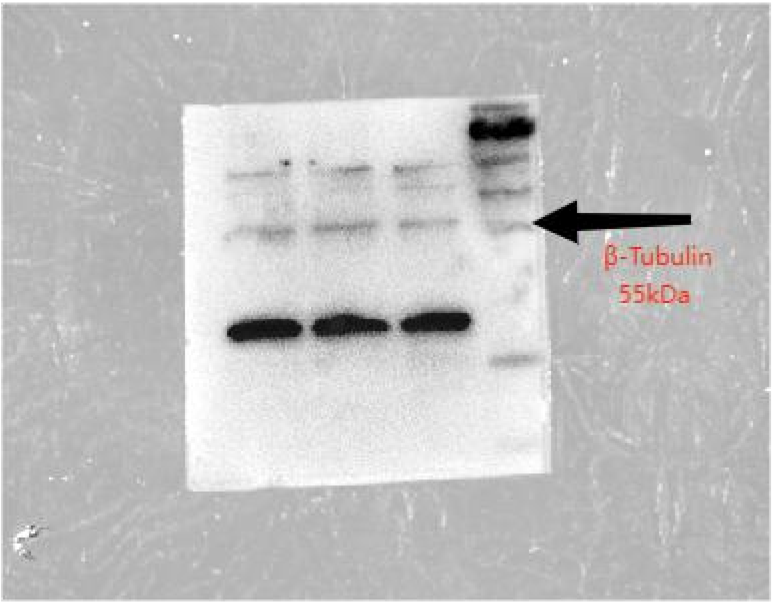

This image is from the same film as NGF. Since the bands of β -Tubulin were not clear, the internal reference images of BDNF were used, provided it had been demonstrated that the internal references of both were neatly.

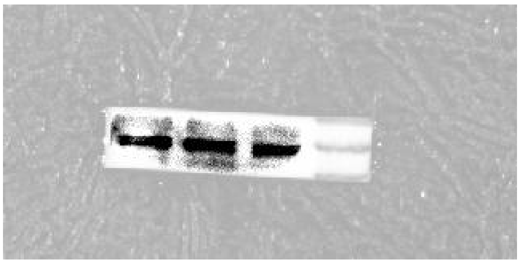
P75NTR,

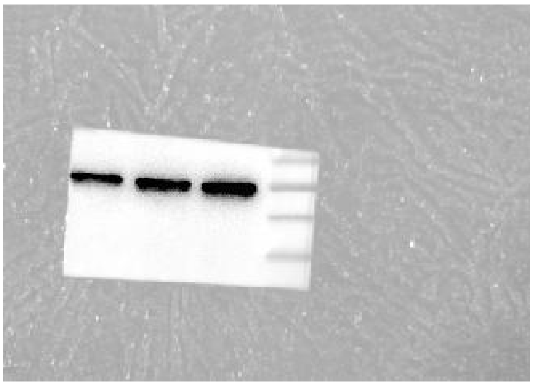
β -Tubulin

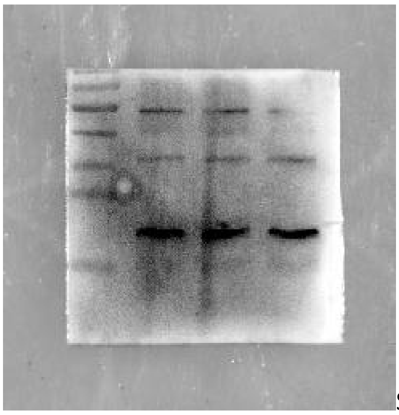
Sox10

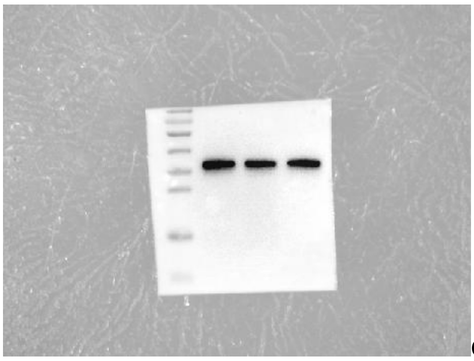
GAPDH
